# A comparative study of aged and contemporary Chinese herbal materials by using delayed luminescence technique

**DOI:** 10.1186/s13020-020-0287-0

**Published:** 2020-01-15

**Authors:** Yusheng Jia, Mengmeng Sun, Yuhua Shi, Zhihui Zhu, Eduard van Wijk, Roeland van Wijk, Tinde van Andel, Mei Wang

**Affiliations:** 1 LU-European Center for Chinese Medicine and Natural Compounds, Institute of Biology, Leiden University, Sylviusweg 72, 2333 BE Leiden, The Netherlands; 2 Institute of Biology, Leiden University, Sylviusweg 72, 2333 BE Leiden, The Netherlands; 3 Naturalis Biodiversity Centre, Darwinweg 2, 2333 CR Leiden, The Netherlands; 4 Changchun University of Chinese Medicine, No. 1035, Boshuo Rd, Jingyue Economic Development District, 130117 Changchun, China; 5 Institute of Chinese Materia Medica, China Academy of Chinese Medical Sciences, Beijing, 100700 China; 6 Meluna Research, Koppelsedijk 1-a, 4191 LC Geldermalsen, The Netherlands; 7 SU BioMedicine, Post Bus 546, 2300 AM Leiden, The Netherlands; 8 Shenzhen Huakai Traditional Chinese Medicine and Natural Medicine Research Center, Shenzhen, 518114 China; 9 State Key Laboratory of Quality Research in Chinese Medicine, Institute of Chinese Medical Sciences, University of Macau, N22 Avenida da Universidade, Taipa, Macau

**Keywords:** Delayed luminescence, Chinese herbal medicine, Aged herbal materials, quality control

## Abstract

**Background:**

Investigation of aged Chinese herbal materials will help us to understand their use and sources in ancient time and broaden the historical perspective of Chinese material medica. To reach this aim, the basic understanding of aged herbal materials, including physical and chemical characters, is of great importance. Delayed luminescence (DL) technique was developed as a rapid, direct, systemic, objective and sample loss-free tool to characterize the properties of Chinese herbal materials. In this study, we measured DL values in aged Chinese herbal materials that were transported from Asia to Europe during the 20th century and stored in Naturalis Biodiversity Center and the Utrecht University museum, and compared these with modern material of the same species.

**Methods:**

A hyperbolic function was used to extract four properties from the DL curves of Chinese herbal material from 1900, the 1950s and recently harvested products. Statistical tools, including the Student’s t test, One-way analysis of variance and Principal Component Analysis, were used to differentiate the DL properties of aged and contemporary collections of *Glycyrrhiza* spp. *Curcuma aromatica* Salisb., *Zingiber officinale* Roscoe, *Alpinia officinarum* Hance and *Acorus calamus* L.

**Results:**

Our results showed that DL properties were significantly different between historical and contemporary Chinese herbal materials. Changes in DL values were species-dependent: the effects of storage time of DL properties were specific for each species. These outcomes help us not only in the identification of historical Chinese medicine products but also provides valuable data of the effect of storage time on herbal materials.

**Conclusion:**

The simple, direct, rapid, and inexpensive measurements offered by DL provide a novel tool to assess the taxonomic identity of Chinese and other herbal materials and assess the differences in chemical properties with increasing storage time. Our results contribute to the further development of novel digital tools for the quality control of herbal materials.

## Background

**Fig. 1:**
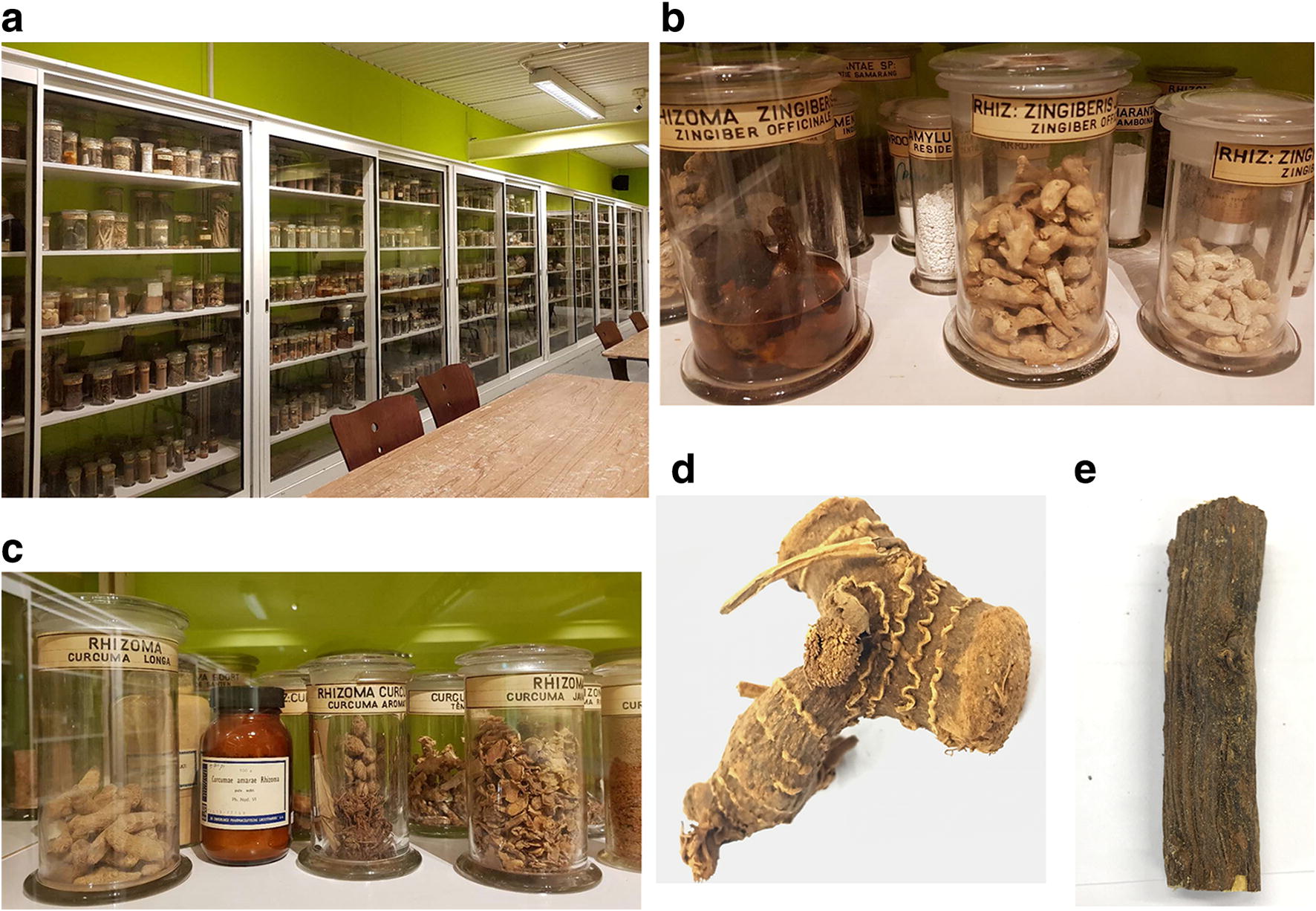
Historic herbal materials used in this study. **a** Display cabinets of historical herbal medicine in Utrecht Botanical Gardens. **b** Historic collections of *Zingiber officinale* (Sample ID Z.o_1900) in Utrecht Botanical Gardens. **c** Historic *Curcuma aromatica* collection (Sample ID C.a_1900) in Utrecht Botanical Gardens. **d** Historic *Alpinia officinarum* rhizome (Sample ID A.o_1900) at Utrecht Botanical Gardens. **e** Historic *Glycyrrhiza glabra* root (Sample ID G.g_1929) in Naturalis Biodiversity Center

Herbal medicine has been used for millennia in China to maintain good health and for the treatment of diseases, and during the last decades it’s global popularity is increasing [1, 2]. Recently, the World Health Organization has included Traditional Chinese Medicine (TCM) in its medical compendium as a recognition of its significant acceptance worldwide [3]. As early as the Chinese Han Dynasty (202 BCE-220BE), the exchange of herbal medicine between China and the outside world has begun through the Silk Road [4]. Around the beginning of the 10th century, Xun Li wrote his work *Extrinsic Materia Medica*, summarizing information on more than 120 herbs introduced into China from abroad [5]. In the same period, due to the gradual development of maritime trade, China began exporting herbal medicines to its surrounding countries and regions. From the Ming Dynasty (1368–1644 AD) onwards, Chinese herbal medicines were shipped to Europe in large quantities through maritime trade, first with Portuguese and later with the Dutch. One of the famous and popular herbal medicines at that time was “China root” (Smilacis Glabrae Rhizoma, the rhizome of *Smilax glabra* Roxb.). It was used to treat syphilis and as a result of increasing global movements and trade, the product became rapidly popular worldwide [6]. From the 17th century onwards, European scientists and explorers collected Chinese herbs progressively for the aim of curiosity, the study of different medical cultures and the interest in exotic medicinal plants. Several European museums and private persons hold collections of historical Chinese herbal medicine, some more than 100 years old, such as the ancient Chinese medicinal material collection in the Natural History Museum in London [7]. Several historic TCM collections are housed by the Utrecht University Museum and Naturalis Biodiversity Center in Leiden in the Netherlands (Fig. 1).

Historic collections of Chinese herbal materials are valuable objects for the scientific study of Chinese culture, trade and ethnopharmacology. Investigation of ancient herbal materials will help us to understand how the use of TCM has changed through history. Centuries ago, the global demand in Chinese herbal materials was not as high as in the present. In the course of time, some of the original plant species were replaced by others. Wild plants are now grown in high production systems [8], while rare plants have gone extinct [9, 10]. For example, Lignum sinensis resinatum, the resinous wood of *Aquilaria sinensis* (Lour.) Gilg, was substituted by Lignum aquilariae resinatum (*Aquilaria agallocha* (Lour.) Roxb.) [11]. According to *Zhonghua Bencao*, *A. sinensis* and *A. agallocha* have similar active constituents and the same clinical effect [12]. In addition, historic local names of herbs have changed or are confused with different plant species with similar names elsewhere in China [11]. All these variations and changes in names and species over time may lead to mistakes in recipes and the use of the wrong herbal materials with potential risks to consumers [13]. Most previous studies that evaluate historical changes in TCM have largely focused on literature research [14], but many of their conclusions have not yet been confirmed by the revision of physical samples from premodern collections of Chinese herbal materials. Therefore, it is critical to have an objective analytic tool for the assessment of these ancient collections of herbal materials [14].

The identification of historic collections of TCM is challenging, as the amount of stored material per species is often very small and fragile. Due to the distinctiveness of these historic collections, we are constrained to perform analytic studies with limited amounts of herbal materials. Instead of destructive methods such as DNA analysis and chemical profiling studies, non-destructive techniques are preferred to identify ancient and very valuable TCM collections. Delayed luminescence (DL) was developed as a rapid, direct, systemic tool to measure the decaying ultra-weak luminescence (up to seconds or minutes) exhibited by materials after being illuminated with light. As a sample loss-free technique, DL is a sensitive approach and widely applied in determining food quality [15], seed germination [16] and cancerous cells [17]. DL is a photo-induced ultra-weak photon emission [18], of which the properties are influenced by molecular structures and interactions [19], in particular the long chain molecules [20, 21]. The molecular absorption of excitation energy determines the dynamics of the subsequent DL emission [19]. Compared with other existing methods, DL has the following advantages: (1) Simplicity. Herbal material only needs be ground to powder, no other complicated treatment is required; (2) Sample loss-free. Powdered medicinal material is exposed to light for 10 s, then the photon released from the sample is recorded by the instrument. There will be no chemical or biological changes in the samples, and they can be used again for other analytical research; (3) Rapidity. The whole experiment process is simple and fast: it only takes several seconds for the measurement and ca. 10 min from sample pre-processing to obtaining the data for one sample; (4) Reduced costs. Compared to chromatography and DNA-barcoding, DL experimental instruments are very cheap and no kits are required for sample preparation.

Recently, various researchers have successfully applied DL in herbal medicine to identify specific properties of herbal materials or to detect variations in the material due to variation in environmental growth conditions [22], different processing methods [23, 24] and determination of authenticity [25]. Differences detected by DL in herbal materials are also reflected by their chemical profiling [24, 26] as well as therapeutic activities [27]. The ability of DL to rapidly distinguish between herbal material with different growth conditions, processing states, taxonomic identity or therapeutic properties, all of which are linked to differences in chemical compositions in the materials, suggest that DL is a promising technology for further evaluation of the quality of herbal material [24, 27].

In this work, we have tested whether DL can also be used to detect changes in herbal medicine over time. We performed DL analysis on historic TCM collections of the species *Glycyrrhiza glabra* L., *Glycyrrhiza inflata* Batalin, *Glycyrrhiza uralensis* Fisch., *Curcuma aromatica* Salisb., *Zingiber officinale* Roscoe, *Alpinia officinarum* Hance and *Acorus calamus* L. and contemporary materials of their corresponding species. The results obtained from our DL measurements show that DL properties indicate differences between aged and contemporary herbal materials, and that DL may be further used to verify the storage time of herbal materials. Therefore, DL can provide new insights into the quality and safety of herbal medicines.

## Materials and methods

### Herbal materials

Historic herbal material was sampled from the collections of Chinese medicine that are housed in the Economic Botany collection of Naturalis Biodiversity Center (Leiden, the Netherlands), which were obtained in the 1950s in Indonesia and from collections of the Utrecht University Museum, stored in the Wachendorffzaal of Utrecht Botanical Gardens (Utrecht, the Netherlands). Due to the small amount of herbal material stored per species, only a limited amount of samples could be taken from the museum collections for this study. Contemporary herbal materials were obtained from the Institute of Chinese Materia Medica, the Beijing Institute of Chinese Medicine, National Institutes for Food and Drug Control and TongRenTang Co., Ltd., all located in Beijing, China. All samples were verified for the correct taxonomic identification by Dr. Mei Wang and Dr. Yuhua Shi and later deposited at the European Center for Chinese Medicine and Natural Compounds of Leiden University (Leiden, the Netherlands).

### Sample preparation and DL measurement

Herbal materials were milled by a grinder (model QE-100, Yili Company, Zhejiang Province, China) and passed through a sieve to obtain 150 μm particles. The powdered herbal material was kept in light-proof boxes containing some 3–5 mm silica gel (BoomBV, Meppel, the Netherlands) at room temperature for 16 h before the DL measurements [23].

DL assays were performed according to the published protocol [23]. The instrument used in our measurements was development by Meluna Research (Geldermalsen, the Netherlands) and included a photomultiplier tube (PMT) (type 9558QB; Electron Tubes Enterprises Ltd., Ruislip, UK), vertically positioned on a dark sample chamber kept at 22 °C. The PMT contained a cathode end (51 mm diameter) with sensitivity at 300–800 nm. The PMT was cooled to − 25 °C in order to reduce the dark count rate to 10 counts per second. A fast preamplifier (model 9301, ORTEC, Oak Ridge, TN) was used to amplify the photon emission signal. Data were extracted by a computer with a model 6602 counting card (National Instruments, Austin, TX). For each sample, 1 g powder was taken and put into a petri dish (diameter: 35 mm), then exposed to light for 10 s using a model 284–2812 white halogen excitation source (Philips, Germany). The DLs of these samples were successively measured three times. The data obtained from the three measurements were used to analyze the DL properties of each sample. The DL decay signature was obtained by recording the number of photon counts in consecutive 0.05-s periods for a total of 60 s, yielding a total of 1200 data points.

### Statistical analysis of DL properties

In order to calculate the DL properties of the samples, all the photon counts measured during the 60 s of each decay curve were used according to the following hyperbolic function [24]:

$$ I_{\left( t \right)} = \frac{{I_{0} }}{{\left( {1 + \frac{t}{Tau}} \right)^{Beta} }} $$

$$ T = \left( {e^{{\frac{1}{ Beta}}} - 1} \right) \times Tau $$

This hyperbolic function is a general formula for fitting the DL decay curve of samples. The four parameters (I0, Beta, Tau and T) obtained by this hyperbolic function can well express the characteristics of the DL decay curve [24, 28]. I0 is the initial intensity of the DL curve, Beta is an index factor associated with the rate of DL decay, and Tau and T represent the DL characteristics and decay time, respectively. The parameters of the repeated measurements (at least three times) were averaged and used to represent the DL properties of each sample. Principal components analysis (PCA) scores were used to indicate the level of discrimination between DL properties by tools provided in the MetaboAnalyst software package (http://www.metaboanalyst.ca). A two-tailed, unpaired Student’s t test and One-way analysis of variance (ANOVA) with least significant difference (LSD) post hoc analysis (SPSS version 23.0) were used to compare the DL properties between herbal samples; differences were considered significant at p < 0.05.

## Results

In order to assess the applicability of DL techniques in detecting differences between aged and contemporary herbal material, five different types of herbal products, each with historical and corresponding recent materials were used in this research. The samples measured in this study are shown in Table 1. The four parameters of the DL decay curves were calculated by a hyperbolic function, which was used to fit the observed decay curves. The differences in four separate parameters were visualized by the PCA, which allowed us to achieve a focused view of the variance in the four properties. The correlation of each parameters to the different samples was illustrated in a PCA biplot.

**Table 1:** Sample information

Pharmaceutical name	Sample id	Scientific name	Sample source	Sampling time
Glycyrrhizae Radix et Rhizoma	G.g_1900	*Glycyrrhiza glabra* L.	Utrecht Botanic Gardens	1900
	G.g_1929	*Glycyrrhiza glabra* L.	Naturalis Biodiversity Center	1929
	G.g_2018	*Glycyrrhiza glabra* L.	Institute of Chinese Materia Medica	2018
	G.i_2018	*Glycyrrhiza inflata* Batalin	Institute of Chinese Materia Medica	2018
	G.u_2018	*Glycyrrhiza uralensis* Fisch.	Institute of Chinese Materia Medica	2018
Curcumae Radix	C.a_1900	*Curcuma aromatica* Salisb.	Utrecht Botanic Gardens	1900
	C.a_1957	*Curcuma aromatica* Salisb.	Naturalis Biodiversity Center	1957
	C.a_2018	*Curcuma aromatica* Salisb.	National Institutes for Food and Drug Control	2018
Zingiberis Rhizoma	Z.o_1900	*Zingiber officinale* Roscoe	Utrecht Botanic Gardens	1900
	Z.o_1952	*Zingiber officinale* Roscoe	Naturalis Biodiversity Center	1952
	Z.o_2017	*Zingiber officinale* Roscoe	TongRenTang Co., Ltd.	2017
Alpiniae Officinarum Rhizoma	A.o_1900	*Alpinia officinarum* Hance	Utrecht Botanic Gardens	1900
	A.o_2017	*Alpinia officinarum* Hance	TongRenTang Co., Ltd.	2017
Acori Tatarinowii Rhizoma	A.c_1900	*Acorus calamus* L.	Utrecht Botanic Gardens	1900
	A.c_2017	*Acorus calamus* L.	TongRenTang Co., Ltd.	2017

**Fig. 2:**
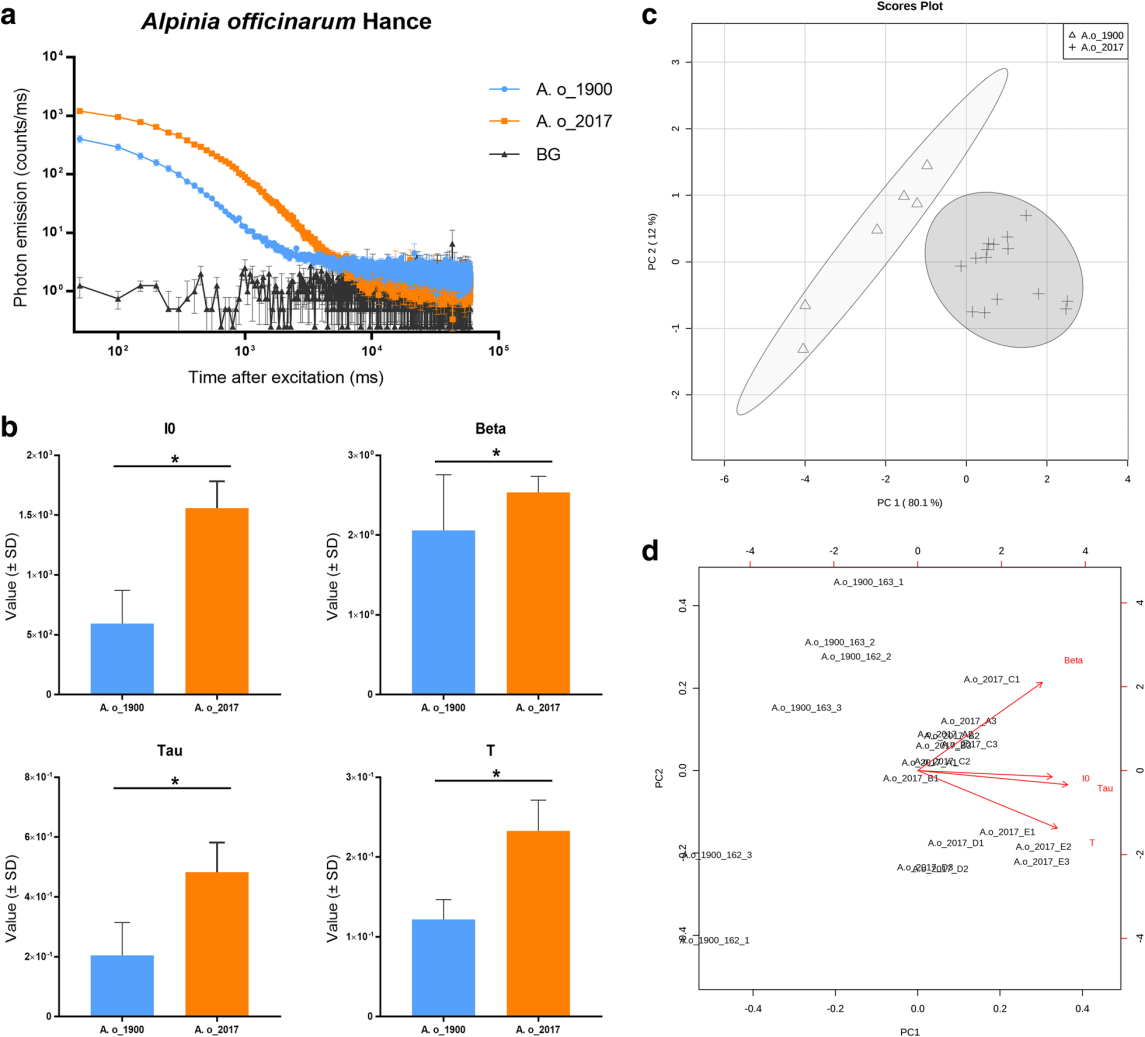
DL analysis of *Alpinia officinarum* Hance samples. **a** DL decay curves comparison among *Alpinia officinarum* Hance samples. BG = background. **b** Comparison of DL properties among the *Alpinia officinarum* Hance samples. I0 is the initial intensity of the DL curve, Beta is an index factor associated with the rate of DL decay, and Tau and T represent the DL characteristics and decay time, respectively. *p < 0.05. **c** PCA score plots of the DL properties obtained from *Alpinia officinarum* Hance samples. **d** PCA biplot indicating how each parameter influences the similarity of DL decay curves

*Alpinia officinarum* and *Acorus calamus* were firstly analyzed. For these two species we had only samples from two different points in time: historical samples from around 1900 and modern samples from 2017. The DL decay curves of *A. officinarum* (Fig. 2a), clearly show differences between samples from 1900 and 2017. The four parameters of the DL decay curve that were compared all differed significantly between the recent and aged samples (Fig. 2b). Figure 2c displays the PCA results in the form of a score plot, in which the *A. officinarum* samples were clustered into two age groups. The PCA biplot (Fig. 2d) reveals that parameters I0, Beta, Tau and T are responsible for distinguishing between the two groups.

**Fig. 3:**
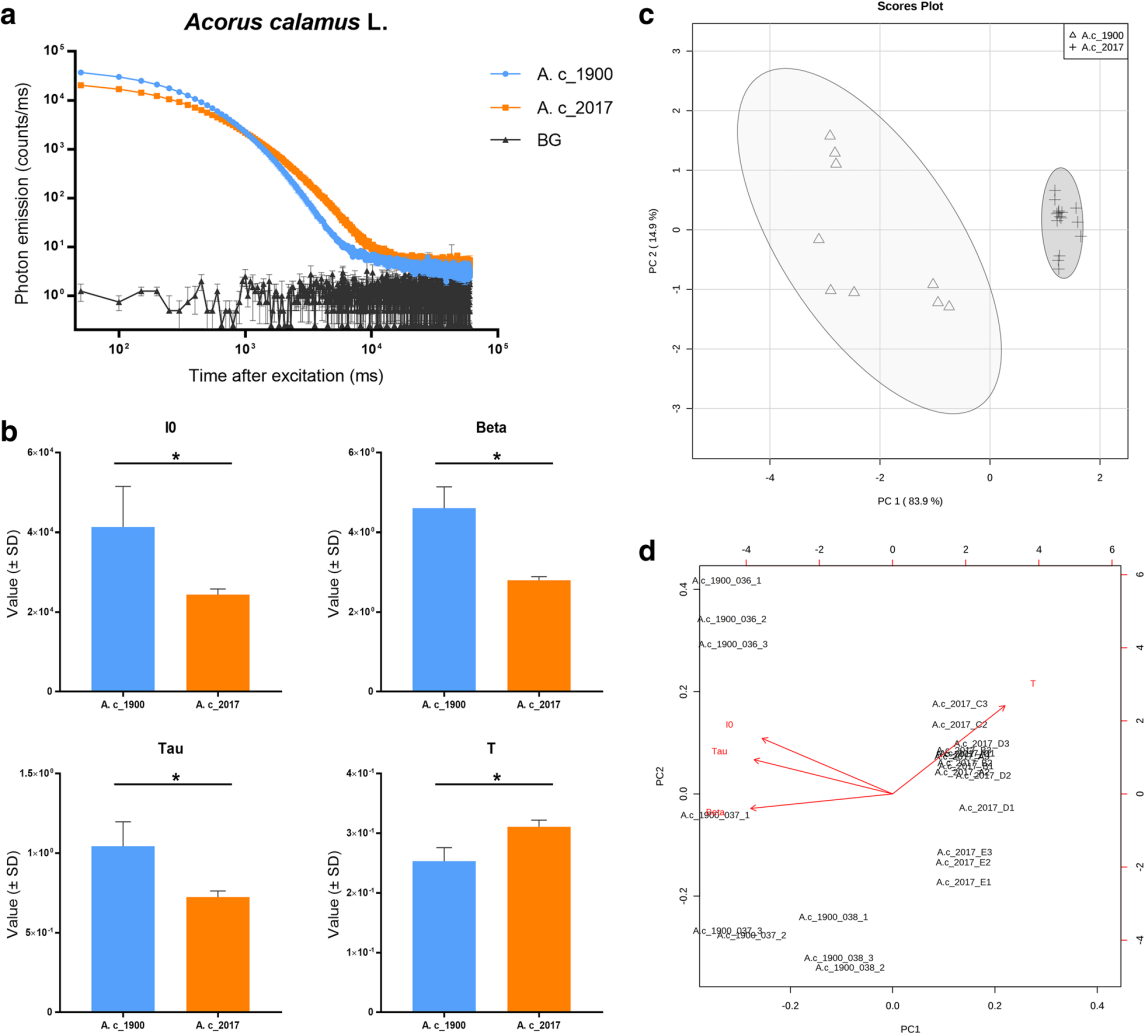
DL analysis of *Acorus calamus* L. samples. **a** DL decay curves comparison among *Acorus calamus* L. samples. BG = background. **b** DL properties comparison among *Acorus calamus* L. samples. I0 is the initial intensity of the DL curve, Beta is an index factor associated with the rate of DL decay, and Tau and T represent the DL characteristics and decay time, respectively. *p < 0.05. **c** PCA score plots of the DL properties obtained from *Acorus calamus* L. samples. **d** PCA biplot shown how strongly each parameter influence the similarity of DL decay curves

The DL decay curve of *Acorus calamus* (Fig. 3a) also shows that samples of different ages have different curves. The DL parameters analysis proved that the initial intensity (I0), curve rate (Beta), DL curve characteristics (Tau) and decay time (T) are significantly different between these two samples (Fig. 3b). The PCA score plot shows that samples of different age clustered into separate groups (Fig. 3c). The PCA biplot indicates that all four parameters contribute to separate samples of different ages (Fig. 3d). The results of our DL analysis of *A. officinarum* and *A. calamus* show that DL technology is capable of discriminating between historic and recently collected herbal materials. All four parameters exhibited significant differences and the PCA clustered samples of unequal ages into different groups. However, as the tested materials were collected in time periods 117 years apart, we wondered whether specimens with less extreme age differences would also differ in their DL properties.

**Fig. 4:**
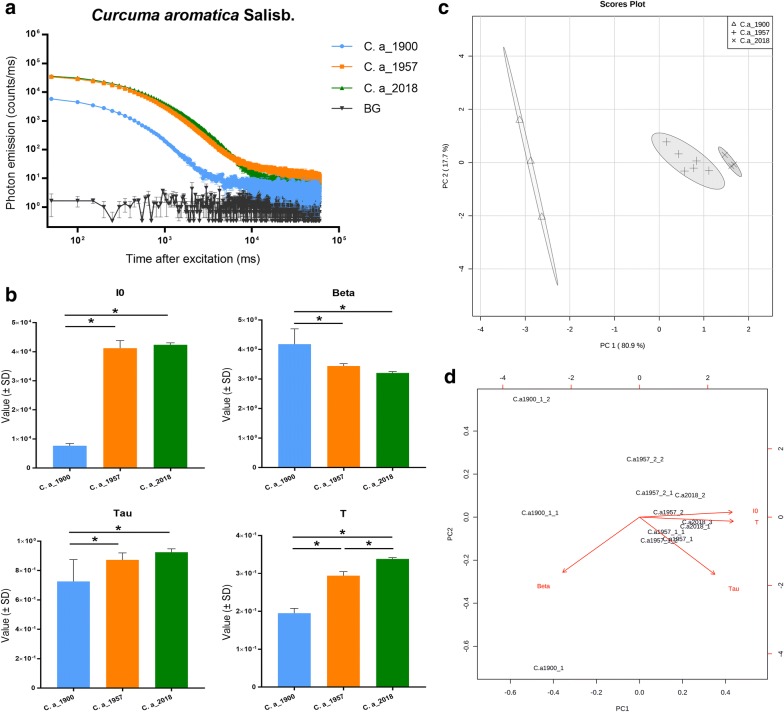
DL analysis of *Curcuma aromatica* Salisb. samples. **a** DL decay curves comparison among *Curcuma aromatica* Salisb. samples. BG = background. **b** DL properties comparison among *Curcuma aromatica* Salisb. samples. I0 is the initial intensity of the DL curve, Beta is an index factor associated with the rate of DL decay, and Tau and T represent the DL characteristics and decay time, respectively. *p < 0.05. **c** PCA score plots of the DL properties obtained from *Curcuma aromatica* Salisb. samples. **d** PCA biplot shown how strongly each parameter influence the similarity of DL decay curves

To answer this question, we used samples of *Curcuma aromatica* and *Zingiber officinale*, which were approximately stored for 60 and 120 years, and compared them to contemporary samples of corresponding species. We subjected the samples to DL analysis to verify whether this technology was able to distinguish three different storage times that were not so far apart. The DL analysis results of *C. aromatica* are presented in Fig. 4. Samples from 1957 and 2018 had similar DL decay curves, while the curve of the sample from 1900 was quite distinctive (Fig. 4a). To compare the four parameters of the DL decay curves, a one-way ANOVA test was used. Results revealed that for four parameters, the sample from 1900 was significantly different from other two more recent samples, while the samples from 1957 and 2018 did not significantly differ except for parameter T (Fig. 4b). The PCA results (Fig. 4c) show that all tested samples were divided into three clusters based on storage time and that the sample from 1900 clustered far from the other two groups. The PCA biplot indicates that parameter I0 and T were heavily responsible for identifying the clusters (Fig. 4d). Together these results suggest that DL technology is able to distinguish samples of *C. aromatica* of 120 years old from 60 year-old samples, but not between 60 year-old and contemporary samples.

**Fig. 5:**
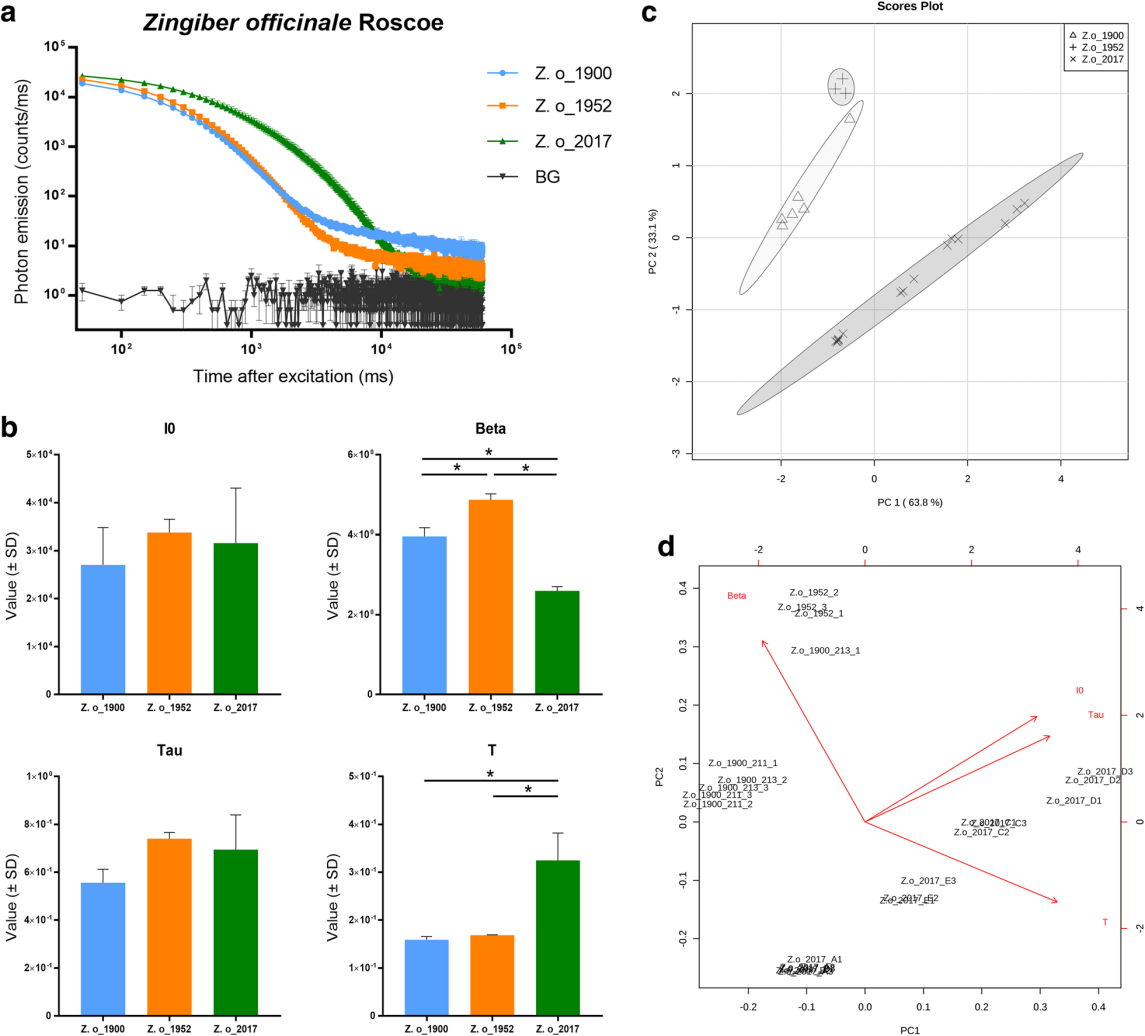
DL analysis of *Zingiber officinale* Roscoe samples. **a** DL decay curves of *Zingiber officinale* Roscoe samples of different ages. BG = background. **b** DL properties of the three different *Zingiber officinale* Roscoe samples. I0 is the initial intensity of the DL curve, Beta is an index factor associated with the rate of DL decay, and Tau and T represent the DL characteristics and decay time, respectively. *p < 0.05. **c** PCA score plot of the DL properties obtained from *Zingiber officinale* Roscoe samples. **d** PCA biplot showing how each parameter influences the similarity of DL decay curves

The DL decay curves for *Zingiber officinale* are shown in Fig. 5, The sample from 2017 was strikingly different than the other two (Fig. 5a). The parameters of the DL decay curves suggest that only parameters Beta and T differ significantly between the samples (Fig. 5b). The PCA results illustrate that samples from 2017 formed a quite distinct cluster, and that the samples from 1900 and 1952 formed two relatively close clusters (Fig. 5c). The PCA biplot demonstrates that the parameters Beta and T were responsible for dividing the samples from each other. These results indicate that DL technology is able to distinguish ginger roots of 120 and 60 years old from relevant contemporary samples, but not between samples of 120 and 60 years old.

For *Curcuma aromatica*, DL analysis showed no significant differences between samples from 1957 and 2018, but both of them significantly differed from the one from 1900. We speculate that 60 years of storage time does not change its DL properties, but 120 years is long enough to do so. Conversely, the DL results of *Zingiber officinale* had no significant differences between samples from 1900 and 1952, but both of them significantly differed from the one from 2017.

Apparently, the DL properties of ginger roots change after 60 years of storage time, but after that they remain stable. The opposite is true for curcuma roots, which apparently start to change in DL properties after ca. 50 years. These findings suggest clearly that time-dependent changes in DL properties of herbal products occur, but that these changes are also species-dependent. Further research with more samples of multiple storage times and more species is needed to verify our speculation.

Apart from changes in DL values over time, we were also interested in the performance of DL technology in distinguishing closely related taxonomic species. Therefore, we compared contemporary and historic samples of Glycyrrhizae Radix et Rhizoma, known as liquorice root, and widely used as medicine, food supplement and flavoring agent. The botanical identity of our historical samples of Glycyrrhizae Radix et Rhizoma was *Glycyrrhiza glabra* L. However, according to the 2015 edition of the Chinese Pharmacopoeia, three different, botanically related species (*G. glabra*, *G. inflata* Batalin and *G. uralensis* Fisch.) can be used as Glycyrrhizae Radix et Rhizoma [29]. Therefore, in our study, two differently aged samples of Glycyrrhizae Radix et Rhizoma (*G. glabra*) and several contemporary samples of Glycyrrhizae Radix et Rhizoma of three different species were used for DL analysis. With samples collected at three distinct points in time and three different species, we continued to verify the capability of DL technology to distinguish among samples of different age and among closely related species.

**Fig. 6:**
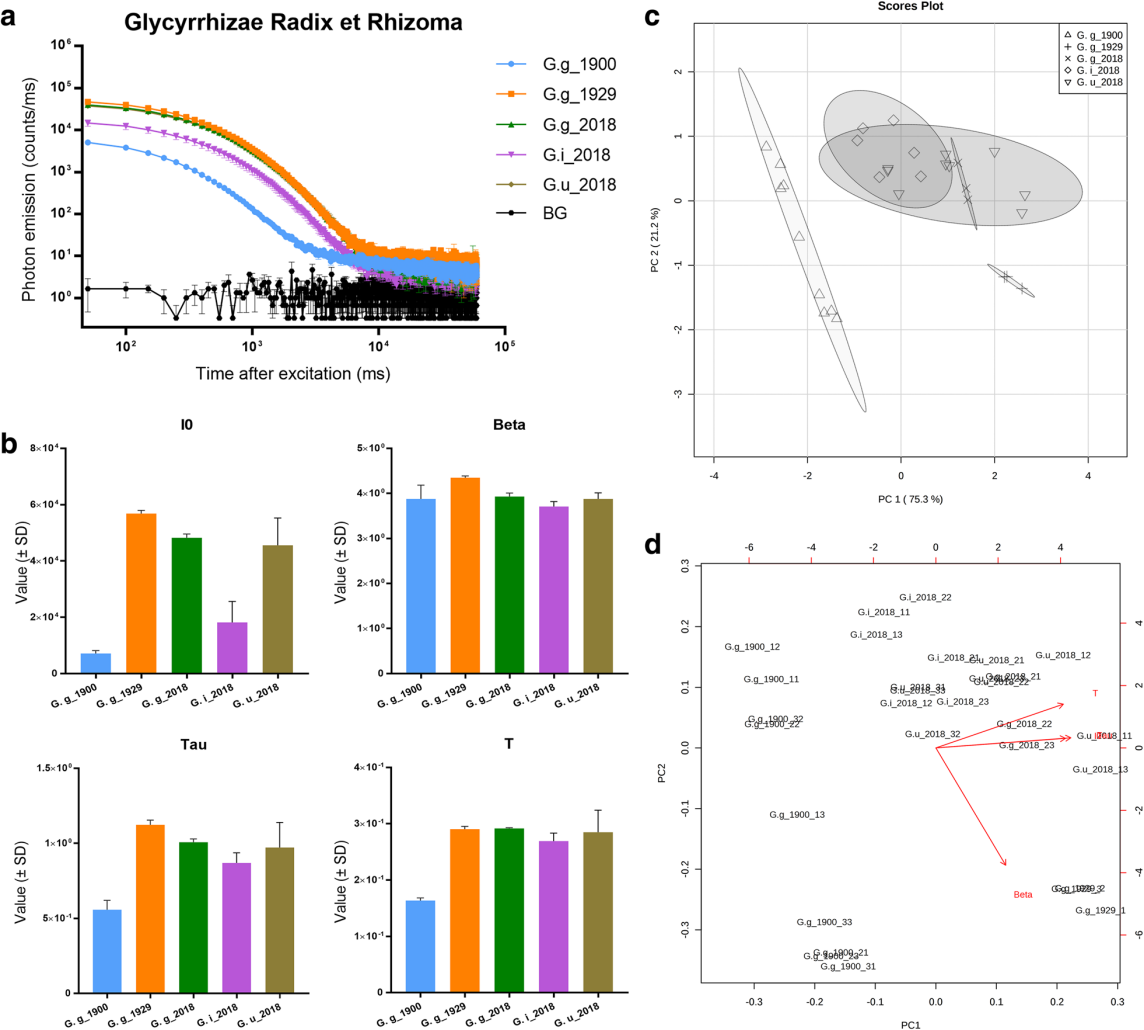
DL analysis of Glycyrrhizae Radix et Rhizoma samples. **a** DL decay curves comparison among Glycyrrhizae Radix et Rhizoma samples. BG = background. **b** DL properties comparison among Glycyrrhizae Radix et Rhizoma samples. I0 is the initial intensity of the DL curve. All five samples’ I0 parameter were significantly different (p < 0.05) from each other, except G.g_2018–G.g_1929 group and G.g_2018–G.u_2018 group. Beta is an index factor associated with the rate of DL decay. The Beta parameter of G.g_1929 was significantly different (p < 0.05) from the other four samples. Tau represent the DL characteristics. The Tau parameter of G.g_1900 and G.g_1929–G.i_2018 group and G.g_1929–G.u_2018 group were significantly different (p < 0.05). T describes the DL decay time. T parameter of G.g_1900 was significantly different (p < 0.05) from the other four samples. **c** PCA clustering of DL properties obtained from Glycyrrhizae Radix et Rhizoma samples. **d** PCA biplot showing how each parameter influences the similarity of DL decay curves

Figure 6 presents the results obtained from the DL analysis of the various samples of liquorice roots. The DL decay curves of G.g_1900 and G.i_2018 are clearly different from the other samples (Fig. 6a), which are all above the background level. Four parameters of the DL decay curves were compared between all samples using a one-way ANOVA test (Fig. 6b). For parameter I0, sample G.g_1900 and G.i_2018 are significantly different from the others. For parameter Beta, sample G.g_1929 differs significantly from the other samples. The Tau parameter of sample G.g_1900 is significantly different as well as between the G.g_1929 and G.i_2018. The T parameter of sample G.g_1900 is significantly different from the other samples. The PCA score plot shows that the Glycyrrhizae Radix et Rhizoma samples are divided into three groups: samples collected in 1900, 1929 and 2018. Interestingly, despite the different species and batches, samples collected in 2018 are clustered into one group. The PCA biplot reveals that parameters I0, Tau and T are mainly responsible for distinguishing group 1900 from the other two groups. Parameter Beta is responsible for distinguishing between group 1929 and group 2018 (Fig. 6d).

Our PCA data illustrate that different aged samples of the same species clustered into different groups, while distinctive species with same collection time showed no significant differences in DL values. Four parameters showed no significant differences, except I0 in sample G.i_2018. PCA clusters were mostly overlapping. Taken together, these results suggest that the DL technique is capable of discriminating samples of different ages of Glycyrrhizae Radix et Rhizoma (at least for *G. glabra*), but not able to discriminate between closely-related species with same collection time.

Glycyrrhizae Radix et Rhizoma is one of the most frequently used herbal products in traditional Chinese medicine. On account of similar active ingredients (glycyrrhizic acid and liquiritin) and clinical effect, the three different species *G. glabra*, *G. inflata* and *G. uralensis* are grouped under the same pharmacological term [29]. Moreover, genetic studies show that the gene sequences of these three species are highly similar [30]. This may be the reason that the DL values of recent samples of these three species are not significantly different. But when taking storage time into account, we do not know whether age has the same impact on DL properties for the three different species. As we only had access to historic samples of *G. glabra*, and not for the other species, we do not know whether DL can distinguish different species of Glycyrrhiza in historic collections.

## Discussion

Investigation of ancient Chinese herbal materials will help us not only to understand the origin and historical use of Chinese herbal medicine, but also to clarify the confusion on botanical identity, nomenclature and changes in species and plant parts over time. Studying historical samples with DL technology gives us new insight into the possible changes in chemical properties of herbal materials over time. Especially when there is a limited amount of herbal material available for testing, DL technique gives us support and solution from an analytic point of view. As DL has already applied to identify different processing methods [24] and determination of authenticity [25], the novelty of the present study is the analysis of aged herbal materials, which provides unique opportunities to understand the effect of long term storage on herbal materials.

In this research, we found that DL can provide sensitive measurements that reflect differences between historic and contemporary herbal materials. DL properties can be affected by changes of molecular conformations and interactions such as forming of hydrogen bonds and carbon-to-nitrogen ratio, resulting in the radiant transfer of energy from one excited molecule to another, causing a change in the material’s DL kinetics. Recently, Grasso et al. [31–33] reported significantly different DL kinetics of intensities and decay time intervals between amylose and cellulose, which share the same glucose-based repeat units, but have differently molecular structures, and the different DL properties of these two compounds may be related to the soliton mechanism. Amylose and cellulose are polysaccharides, which occur widely in herbal material as bioactive components, such as liquorice roots (*Glycyrrhiza* spp.) [34]. With increasing storage time, the polysaccharide content in plants may change, causing a change of molecular structure [35]. Large time intervals may cause significant changes in the chemical composition of polysaccharides. This may be the reason that DL distinguished historic and modern samples of Glycyrrhizae Radix et Rhizoma, but not the three botanically different *Glycyrrhiza* samples collected in similar periods.

The bioactive components in the other medicinal species used in this research are mainly volatile oils [29]. The volatile oil content of herbal material also changes during the storage time [36], which may be the reason why the DL characteristics of our long-term stored samples were so different from recently harvested samples. Future studies should focus on the chemical differences between historical and contemporary herbal material to investigate whether DL could characterize the chemical changes caused by storage time.

Storage time is an important factor in the stability of plant products and thus the quality of herbal medicine. Storage usually modifies the composition of herbal medicines, directly affecting safety and therapeutic value [35, 36]. Most methods used to study composition changes caused by storage time are HPLC or GC–MS [37], but these require expensive analytical tools and sample loss is inevitable. Compared to chromatographic methods, DL is a direct, rapid approach, which is quite affordable and does not imply sample loss. Moreover, DL can provide a comprehensive perspective for the sample’s overall features, rather than only measuring the amount of certain components [27]. Therefore, DL maybe a suitable technology to detect changes in herbal material influenced by storage time. In addition, due to distinctiveness and limited amounts of ancient herbal materials, DL is suitable to study the differences between ancient and contemporary herbal material of the same species. Because of DL’s sample loss-free nature, it is suitable as the first analytic method, so it can be decided afterwards if it is necessary to employ further destructive methods such as DNA analysis [38] and chemical profiling studies. Our study has shown that DL has the potential as a practical approach to verify the applicability of herbal material for clinical use. However, we need to establish a significant database for this purpose. In general, herbal material that has been stored for decades or centuries is no longer suitable for clinical use, but may provide valuable information to understand (changes in) properties of the material. To examine this possibility, using herbal materials with different storage time (e.g., from 1 to 5 years) and combining DL, chemical study and bioactivity analysis will provide a better understanding of changes in herbal material over time.

## Conclusions

In this study, we used historical Chinese herbal medicine as research object to verify whether Delayed Luminescence was a suitable technology to analyze the differences between aged herbal material and corresponding contemporary herbal material. Our findings suggest that DL is a promising approach tool to study historical herbal material, as it is able to identify different properties among samples with different storage time. Our study also showed that patterns of properties changes are likely to be plant species dependent. Our study contributes to the lack in scientific data on the effects of storage time on herbal material. More research should focus on expanding the number of herbal species for DL analysis, data accumulation and mining to better understand DL technology’s potential on assessment of quality related aspects of herbal material.

## Data Availability

The datasets used in this study are available from the corresponding author upon reasonable request.

## Abbreviations

DL: delayed luminescence
PCA: principal component analysis
ANOVA: analysis of variance

## References

[1] He M, Huang X, Liu S, Guo C, Xie Y, Meijer AH (2018). The difference between white and red ginseng: variations in ginsenosides and immunomodulation. Planta Med.

[2] Scheid V (1999). The globalisation of Chinese medicine. Lancet.

[3] Cyranoski D (2018). Why Chinese medicine is heading for clinics around the world. Nature.

[4] Heyadri M, Hashempur MH, Ayati MH, Quintern D, Nimrouzi M, Mosavat SH (2015). The use of Chinese herbal drugs in Islamic medicine. J Integr Med-Jim..

[5] Zhang L (2013). Li Xun’s Oversea Materia Medica and the effect of Islamic culture on TCM. J Pract Trad Chin Intern Med.

[6] Winterbottom AE (2014). Of the China root: a case study of the early modern circulation of materia medica. Soc Hist Med.

[7] Zhao ZZ, Zhao KC, Brand E (2015). Identification of ancient Chinese medicinal specimens preserved at Natural History Museum in London, China. J Chin Materia Med.

[8] Wang J, Sasse A, Sheridan H (2019). Traditional chinese medicine: from aqueous extracts to therapeutic formulae. Plant Extracts.

[9] Chen SL, Yu H, Luo HM, Wu Q, Li CF, Steinmetz A (2016). Conservation and sustainable use of medicinal plants: problems, progress, and prospects. Chin Med.

[10] Hamilton AC (2004). Medicinal plants, conservation and livelihoods. Biodivers Conserv.

[11] Zhao Z, Yuen JP, Wu J, Yu T, Huang W (2006). A systematic study on confused species of Chinese materia medica in the Hong Kong market. Ann Acad Med Singapore.

[12] Committee Zhonghua Bencao Edit (1999). Zhonghua Bencao.

[13] Lord GM, Cook T, Arlt VM, Schmeiser HH, Williams G, Pusey CD (2001). Urothelial malignant disease and Chinese herbal nephropathy. Lancet.

[14] Brand E, Leon C, Nesbitt M, Guo P, Huang R, Chen H (2017). Economic botany collections: a source of material evidence for exploring historical changes in Chinese medicinal materials. J Ethnopharmacol.

[15] Chen W, Xing D (2005). Rapid detection of *Aspergillus flavus* contamination in peanut with novel delayed luminescence spectra. Photochem Photobiol.

[16] Costanzo E, Gulino M, Lanzano L, Musumeci F, Scordino A, Tudisco S (2008). Single seed viability checked by delayed luminescence. EBJ.

[17] Scordino A, Campisi A, Grasso R, Bonfanti R, Gulino M, Iauk L (2014). Delayed luminescence to monitor programmed cell death induced by berberine on thyroid cancer cells. J Biomed Opt.

[18] Scordino A, Baran I, Gulino M, Ganea C, Grasso R, Niggli JH (2014). Ultra-weak delayed luminescence in cancer research: a review of the results by the ARETUSA equipment. J Photochem Photobiol B.

[19] Barenboǐm GM, Domanskiǐ AN, Turoverov KK (2013). Luminescence of biopolymers and cells.

[20] Li X, Baryshnikov G, Deng C, Bao X, Wu B, Zhou Y (2019). A three-dimensional ratiometric sensing strategy on unimolecular fluorescence-thermally activated delayed fluorescence dual emission. Nat Commun..

[21] He M, Sun M, Koval S, Van Wijk R, Hankemeier T, Van der Greef J (2019). Traditional Chinese medicine-based subtyping of early-stage type 2 diabetes using plasma metabolomics combined with ultra-weak photon emission. Engineering..

[22] Sun M, Li L, Wang M, van Wijk E, He M, van Wijk R (2016). Effects of growth altitude on chemical constituents and delayed luminescence properties in medicinal rhubarb. J Photochem Photobiol B.

[23] Sun M, van Wijk R, van Wijk E, Wang M, van Wietmarschen H, Hankemeier T (2016). Delayed luminescence: an experimental protocol for Chinese herbal medicines. Luminescence.

[24] Sun M, Chang WT, Van Wijk E, He M, Van Wijk R, Wang M (2018). Application of delayed luminescence method on measuring of the processing of Chinese herbal materials. Chin Med..

[25] Sun M, Wang S, Jing Y, Li L, He M, Jia Y (2019). Application of delayed luminescence measurements for the identification of herbal materials: a step toward rapid quality control. Chin Med..

[26] Sun M, He M, Korthout H, Halima M, Kim HK, Yan Y (2019). Characterization of ginsenoside extracts by delayed luminescence, high-performance liquid chromatography, and bioactivity tests. Photochem Photobiol Sci.

[27] Sun M, Chang WT, Van Wijk E, He M, Koval S, Lin MK (2017). Characterization of the therapeutic properties of Chinese herbal materials by measuring delayed luminescence and dendritic cell-based immunomodulatory response. J Photochem Photobiol B.

[28] Pang J, Yang M, Fu J, Zhao X, van Wijk E, Wang M (2016). Classification of Chinese herbs based on the cluster analysis of delayed luminescence. Luminescence.

[29] Chinese Pharmacopoeia C (2015). The Pharmacopoeia of the People’s Republic of China, 2015 Edition Part I.

[30] Kondo K, Shiba M, Yamaji H, Morota T, Zhengmin C, Huixia P (2007). Species identification of licorice using nrDNA and cpDNA genetic markers. Biol Pharm Bull.

[31] Grasso R, Musumeci F, Triglia A, Brizhik L, Scordino A (2019). Impact of structure on the delayed luminescence of d-Glucose-based polymer chains. J Photochem Photobiol B.

[32] Brizhik L, Musumeci F, Scordino A, Triglia A (2000). The soliton mechanism of the delayed luminescence of biological systems. Europhys Lett.

[33] Brizhik L, Scordino A, Triglia A, Musumeci F (2001). Delayed luminescence of biological systems arising from correlated many-soliton states. Phys Rev E: Stat Nonlin Soft Matter Phys.

[34] Wei L, Xin-bo S, Cheng-rong S, Qing X (2013). Content determination of polysaccharides in radix glycyrrhizae from three different species. Tianjin J Trad Chin Med.

[35] Zhang J (2016). Comparison in bioactivity of polysaccharide fractions from Fuzhuan brick-tea at different storage periods.

[36] Wu S, Jiang H, Zhang F, Chen W, Jin J (2015). The effect of processing methods and storage time on the volatile oils of *Zanthoxylum armatum* v novemfolius in Jiangjin, Chongqing Province. China Condiment.

[37] Martinazzo AP, Melo EC, Barbosa LCD, Soares NDF, Rocha RP, Randuz LL (2009). Quality parameters of cymbopogon citratus leaves during ambient storage. Appl Eng Agric.

[38] Han JP, Pang XH, Liao BS, Yao H, Song JY, Chen SL (2016). An authenticity survey of herbal medicines from markets in China using DNA barcoding. Sci Rep.

